# Sports Teaching, Traditional Games, and Understanding in Physical Education: A Tale of Two Stories

**DOI:** 10.3389/fpsyg.2020.581721

**Published:** 2020-09-25

**Authors:** Raúl Martínez-Santos, María Pilar Founaud, Astrid Aracama, Asier Oiarbide

**Affiliations:** ^1^Department of Physical Education and Sports, Faculty of Education and Sport, University of the Basque Country, Vitoria-Gasteiz, Spain; ^2^Department of Didactics of the Musical, Plastic and Corporal Expression, Faculty of Education, University of Zaragoza, Zaragoza, Spain; ^3^Department of Physical Education and Sports, University of the Basque Country, Vitoria-Gasteiz, Spain

**Keywords:** traditional game, physical education, philosophy of sport, learning transfer, motor praxeology, TGfU, semiotricity

## Abstract

Unlike Dickens’s novel, this is not a tale of light and darkness, order and chaos, good and evil…It is, though, a story worth to be told about two standpoints about games and sports, teaching and research, physical education simply put, that have pursued similar interests on parallel tracks for too long, despite their apparent closeness and shared cultural grounds. The objective of this conceptual analysis is to try and reconcile two perspectives, namely motor praxeology and teaching games for understanding (TGfU), born in the last third of the XX century in France and England with the intention to rethink the foundations of physical education (PE) and sports teaching. Pierre Parlebas, from the French side of the English Channel, claimed in 1967 that sports make part of PE, that team sports must be considered from a specific, sociomotor point of view, and that motor conducts (i.e., the significative organisation of motor behaviour), not sports techniques, are the corner-stone of PE and sports coaching. In the early 1980s, from the English side of La Manche, Almond, Thorpe, and Bunker made a plea for a shift in the way to teach games (sporting collective duels mostly), deeply concerned by the negative impact of the traditional technics-centred approach on motivation, competence and attained level of the least able in school situations. Our conclusion is that TGfU, or game-based approaches to sports coaching and teaching, can take great advantage of the motor-praxeological rationale for three reasons: firstly, because concepts like understanding, game sense and action principles are operatively, semiotically linked to the reality of the playing process; secondly, because the inner structures of the games that constrain players and guide their motor conducts, permit to integrate games in the general system of sporting games, no matter their level of institutionalisation; finally, because any motor intervention process is better thought of and more systematically developed upon the operational concepts of internal logic and expected practical effects of game playing. This time, Paris could be the place to go to in search of solutions, not the city to run away from in hope of consolation.

## Games, Traditions, and Physical Education

Traditional sporting games, those activities collected by Brueghel the Elder (1560), Stella (1657); Gomme (1894), and Grupos Etniker Euskalerria (1993), constitute a major asset for physical education (PE) in three ways at least: *epistemologically*, they allow us to think about what human action and motricity are in relation to culture, history, and society; *pedagogically*, they let us consider what our options are when proposing aims and designing curricula; and *didactically*, they impel us to question what our resources can be when teaching in PE and sports. The troubled waters of the 1960s, a decade that started with the fall of an “iron curtain” and finished with the rise of humankind to the moon, also stirred the way games and sports were considered on both sides of the English Channel in regard to PE. In this sense, we intend to reflect on those three topics while trying to find an answer to a question we have put some thought on lately: Why are traditional games absent from the Teaching Games for Understanding (TGfU) rationale?

In *mai 68*, another revolution was *en marche* in the Midlands that would definitively affect for the better the way that hundreds of thousands of pupils around the world confront games and sports at schools. It is well known how Thorpe et al. (1986; Sánchez-Gómez et al., 2014) gathered in Loughborough a group of PE teachers, lecturers, and scholars concerned by the way *games* were being used in primary and secondary schools. Over time, that project developed by “the games team” became one of the most recognisable currents in contemporary PE and an inspiration for interesting academic debates, no matter how close they may be to the original project (Kirk, 2017). Presented originally by Thorpe and Bunker as a model for teachers, TGfU’s six phases of instruction is a fruitful combination of pedagogical concerns, didactical needs, and theoretical reflections that harnessed and channelled the interest of many PE professionals in the English-speaking world. On his part, Pierre Parlebas spent *the Beatles decade* in Paris lecturing and training Olympic athletes at the *École Normal Supérier d’Éducation Physique* (Parlebas, 2014) and getting a degree in psychology and other minor qualifications in sociology, linguistics, and mathematics to develop the project of a scientific physical education sketched in his first, visionary paper: *Éducation physique et éducation philosophique* (Parlebas, 1959). Twenty-five years later, he successfully applied for the position of professor at the Faculty of Sociology of *La Sorbonne*, after having obtained at it his state doctorate claiming, in front of an egregious panel, that “sporting game and motor action belong to a specific field of research endowed with an original scientific pertinence” (Parlebas, 1985b, p. 90). In proving so, he has given us the conceptual and methodological resources to act and reflect on PE in the form of a scientific framework, and enough evidence of the richness and importance of motor traditional games for school pupils, university students, PE practitioners, and coaches alike (Parlebas, 1985a, 1998, 2001, 2003a,b, 2010a,b).

Unlike Dickens’s novel, this is not a tale of light and darkness, order and chaos, good and evil… It is, though, a story worth to be told about two perspectives on games and sports, on teaching and research, on physical education simply put, which may have been inspired by similar aims, but driven on parallel tracks for too long despite their geographical closeness and shared historical backgrounds. Therefore, our main objective is to try and articulate two perspectives, namely, “teaching games for understanding” (Thorpe et al., 1986) and “motor praxeology” (Parlebas, 2013), both born in the last third of the twentieth century to *rethink* the foundations of physical education (PE) and sports teaching. It is somehow a *what if* kind of story in the vein of Stolz and Pill (2016), a fictional game sometimes, but not useless nor stupid: *What would TGfU be like today if Thorpe, Bunker, Almond, and Parlebas had met at the right time?* As we hope to prove, this is a pertinent search of conceptual clarification and mutual enrichment in which those humble, youthful traditional sporting games play a key role when looking into three main questions of PE and TGfU: what those activities we call games are, what the consequences of their inclusion in PE can be, and what the principles for their teaching should be.

## On Game’s Definition and Games’ Categories

*Game* and *sport* are extremely polysemic words. Probably not by chance did Wittgenstein chose “game” to illustrate his theory of meaning: “Consider for example the proceedings that we call ‘games.’ I mean board games, card games, ball games, Olympic games, and so on. What is common to them all? – Don’t say: ‘There *must* be something common, or they would not be called *games*’ – but *look and see* whether there is anything common to all. – For if you look at them you will not see something that is common to *all*, but similarities, relationships, and a whole series of them at that” (1953: Wittgenstein, 1963, p. 31). Wittgenstein claimed that we can use a word properly without being able to produce its proper definition, as we do with numbers. However, competent use of a word is just the first level of *clarity* we can attain, as Charles S. Peirce believed: “Merely to have such an acquaintance with the idea as to have become familiar with it, and to have lost all hesitancy in recognising it in ordinary cases, hardly seems to deserve the name of clearness of apprehension” (Peirce, 1878, p. 2). Peirce, possibly the greatest North American philosopher, identified *definitions* as the second level of clarity: “By the *contents* of an idea logicians understand whatever is contained in its definition. So that an idea is *distinctly* apprehended, according to them, when we can give a precise definition of it, in abstract terms” (Peirce, 1878, p. 3).

TGfU is a real academic endeavour (Werner and Almond, 1990; Hopper and Bell, 2001), but it is hard to find in its vast literature a proper discussion about what is understood when the word “game” is uttered in this field, no matter how consistent the use of the word “game” may be. The members of the *games team* were committed to the practice of PE but did not lack influence and inspiration by key academics, such as the philosopher Bernard Suits, the educational thinker Lawrence Stenhouse, and the psychologist Jerome Bruner (Harvey et al., 2018). Precisely, Bernard Suits, whose *What is a game?* (1967) and *The Grasshopper* (1978) are still today fruitful in the philosophy of sport, has been acknowledged as a major influence in the construction of TGfU, which makes this apparent absence of reflection on the nature of game even more intriguing, and certainly due to something else than lack of awareness or interest. It looks like the early authors of TGfU did not need to define *what a game is* because everybody knew *what we are talking about*: “Physical activities using an object that are played in society, for example football, tennis, golf, and softball” (Hopper and Bell, 2001, p. 14), or, as Mauldon and Redfern said in their *Games teaching:* “An activity in which a minimum of two people, themselves on the move, engage in competitive play with a moving object within the framework of certain rules” (Mauldon and Redfern, 1969, p. 1).

Nonetheless, the inquiry on the nature of game-playing is as controversial as helpful for PE thinkers in terms of curriculum building and teaching practice because it is not a simple one: Suits declared that “play, game, and sport” are a *tricky conceptual triad* (1988) when it comes to characterising different ludic experiences and activities, so tricky as to lead him and his contender Meier (1988) to commit in Schneider’s opinion a “category mistake” (Schneider, 2001, p. 151). The Gordian problem of game’s definition was resolved by Parlebas with a clear-cut, new referent: “For us, a sporting game is any motor situation of regulated confrontation, so-called ‘game’ and ‘sport’ by social organisations. […] Certainly poor regarding its notional content, this definition serves mainly identifying purposes, pointing at repertoires of practices proposed by federal or educational organisations” (Parlebas, 1986, p. 46), and leading to some interesting *logical* consequences: board games, like chess, are not sporting games; sporting games are legal entities that regulate human motor action; and playing tags and playing football belong to the same category of “sporting game,” no matter their level of institutionalisation.

**FIGURE 1:**
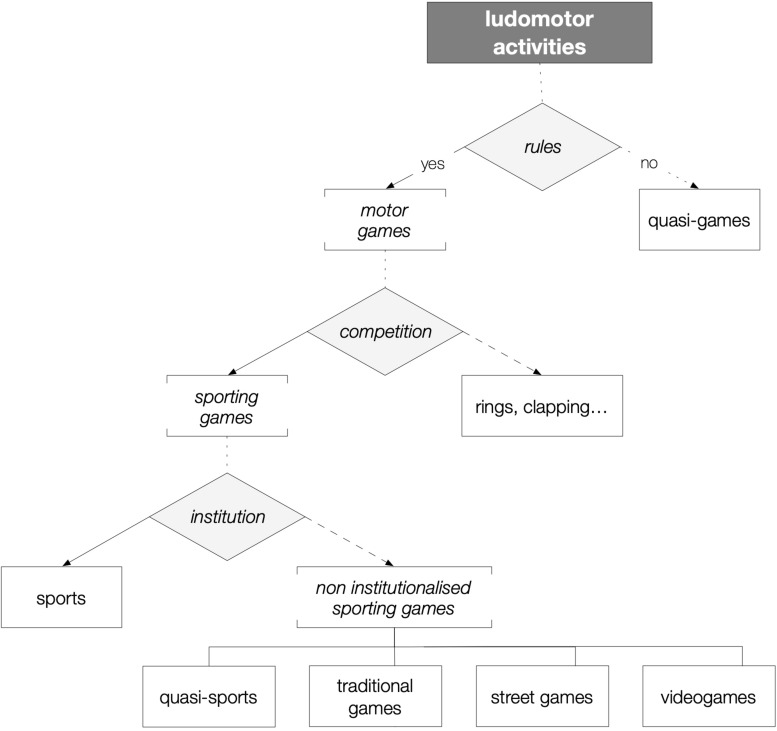
System of categories of ludic motor situations (Parlebas, 2008).

As shown in Figure 1, “ludomotricity” (Parlebas, 1999, L:56)^1^, the total of situations associated with playful motor situations, can be categorised using a few but distinctive traits, giving as a result what Parlebas described as a *spectacular ludorama* (Parlebas, 2008):

• Swimming, jogging, skating, etc., are free, self-organised actions that impose no restriction whatsoever on the agents, reason why they cannot be properly called games, but “quasi-games.”
• Hand clapping games, like *My mummy sent me shopping* and *A sailor went to sea*, or more recent games like *the cup challenge*, are the minimum expression of the class “motor game,” that is, activities that impose on the players prefixed bodily behaviours that can be valued accomplished or not without establishing a true competition, that is, without calls by referees or umpires.
• Athletics, football, tennis, and the likes are “sports”: institutionalised motor competitions, sporting games with constitutive rules for players and roles for rules-making and disciplinary action.
• The largest part of “sporting games” are not institutionalised, or they are administrated by local or regional governing bodies: in this case, we can call them “quasi-sports” (i.e., Highland games or traditional bowling games), or “traditional games” when they show no level of organisation at all (i.e., hide-and-seek, tags, etc.). Besides, “street games,” like self-organised basketball (Bordes et al., 2013), and certain “videogames,” the so-called exergames, for instance, belong to this ample category too.

This system of ludomotor categories addresses two capital questions on games and PE: their motor nature and the different levels of constriction that operate on the players. *Motor situations* constitute a special case of Goffman’s *situation*: “I would define a social situation as an environment of mutual monitoring possibilities, anywhere within which an individual will find himself accessible to the naked senses of all others who are ‘present,’ and similarly find them accessible to him. According to this definition, a social situation arises whenever two or more individuals find themselves in one another’s immediate presence, and it lasts until the next-to-last person leaves” (Goffman, 1964, p. 135). What makes them special is that both the process and the result of a ludomotor situation depend on the bodily configuration of the agents’ behaviours, which always are connected to the overall meaning of the encounter. For this reason, any element of the situation receives “motor” as a family name because their bodily, physical nature is the essence of this unique kind of experience that characterises PE.

Swimming or climbing, hand-clapping or playing tags, fishing of racing, and playing soccer or rugger are all *motor actions* consisting of processes “of accomplishment of the motor conducts of one or more individuals acting in a determined motor situation” (Parlebas, 1999, A:1), embodiments of a kind of human action that owns very specific properties (Bordes et al., 2007): it is constrained by the physical and biological laws that operate on individuals, species, and material world; it is constitutive to the task to perform, which disappears if the action is not accomplished (means and results are equally necessary); and it is real action, not symbolic (like in board games) nor substitutive (like in competitive videogames). Game-playing is meaningful in itself and independent of its technical sophistication or skill development: the Sunday round of four pals over handicap 20 makes so much sense for the players as the afternoon singles in the last Ryder Cup, and playing darts is as bodily an action as figure-skating.

Having said so, sporting games are not only *physical* but also *cultural realities* independent of what agents can think they are. Sporting games are linguistic, juridical entities that create specific action spheres easily transmitted by word (Martínez-Santos, 2018). TGfU pays paramount attention to “rules”: linguistic utterances whose function is to orient and regulate human action (Robles, 1984), because not only games are created by rules, but teaching activities too. The capability to play games relies on a general linguistic competence that makes games interpretable and transformable into bodily conducts, which is the same competence that allows teachers to *make understand* the game (Martínez-Santos and Oiarbide, 2020). Briefly put, rules are *necessary* to decree the elements of the game, that is, to create competition: the accepted motor procedures and the consequences associated with acts and results. “Consequences” are, in fact, the most important part of these juridical systems we call games: on one side, they value acts and keep a record of the players’ merit to outperform; on the other side, they establish how agents must proceed in case of infringement. Any moral value of game playing depends on the acceptance of its internal consequences and the development of the juridical intelligence that wraps tactical awareness, which in the games we are dealing with is based on the “principle of sanction” (Robles, 1984): rule-breaking has as consequence damage that sometimes affects one’s score (i.e., volleyball and judo) and some others does not (i.e., basketball and hockey). Moreover, this juridical nature allows circumventing the *annoying* thesis of the “logical incompatibility” (Morgan, 1987) between breaking the rules and the existence of the game.

It may seem a minor question, but these three criteria, namely, motricity, regulation, and institutionalisation, help us mitigate Wittgenstein’s blurriness and advance in Peircean clarity when it comes to exploring *physical* activities. Sports are institutionalised sporting games that “have been selected and consecrated by social institutions that have put them into their structures of production and consumption” (Parlebas, 1986, p. 46). There is no doubt that *games* in TGfU are motor competitions, but it seems very clear as well that they are *only* competitions managed by international governing bodies, and there is a chance that sports federations and school institutions have different understandings of what *production and consumption* are or should be.

## On Games in Physical Education

Sports are a tradition in PE. We are so accustomed to their presence that it is felt natural, inevitable, and indisputable. However, as we have learnt above, acquaintance is just the dimmest level of clarity, and conceiving PE as school sports practice would be no definition but a mere description of the research object we would eventually like to understand. Peirce translated the third and highest level of clarity into his famous *pragmatic maxim*: “Consider what effects, which might conceivably have practical bearings, we conceive the object of our conception to have. Then, our conception of these effects is the whole of our conception of the object” (Peirce, 1878, p. 20). Our educational decisions, theoretical classification systems, conceptual or empirical diagrams, hypothesis and beliefs, etc., are examples of *practical bearings* that Peirce considered to be signs of intelligence in action. In this sense, understanding what sporting games are also depends on discussing why they belong to education, firstly, and what the consequences of our including sports in PE are, secondly.

In a world as utopian as *The Grasshopper’s* kingdom imagined by Bernard Suits (Suits, 1978), The Monty Python comedy troupe depicted a British government with a *Ministry of Silly Walks*. This memorable sketch (The Monty Python, 1970) depicts an applicant trying to show and prove that his gait is as silly as to deserve public funding, and a public servant analysing his merits in a very professional way: “It’s not particularly silly, is it? I… I mean, the right leg isn’t silly at all, and the left leg merely does a forward aerial half-turn every alternate step.” The postulant’s initial disappointment turns into joy when he is offered “a research fellowship on the Anglo-French silly walk”: *La marche futile!* Isn’t it possible that to many a *ministry of PE and sports* be as comical and useless as the one created by The Monty Python, apparently to criticise the Concorde’s project? Maybe, but it is almost sure that to many, and not only to those fans standing on the bleachers, traditional sporting games are childish, minor games in comparison to pay-per-view sports. However, quite on the contrary, there could be a point in thinking that any uncritical inclusion of sports artificial techniques in PE might reduce any allegedly educational game to nothing more than another silly walk in search for a generous, even sillier public servant to be turned into a fan.

### On Sports and Physical Education

“To those advocates of TGfU derivates that seek to produce excellent games players in specific sports coaching contexts, such as Games Sense, Bunker and Thorpe were not, at least originally, ever concerned with sports coaching pedagogy. For those who claim that TGfU emerged without a substantial theoretical framework, the problem Bunker and Thorpe were seeking to resolve was practical and pedagogical, concerned with institutional school physical education.” He who reminds us of this is Kirk (2017, p. 19), deeply interested in making clear that “TGfU-informed games teaching was intended to fit into same spaces that sports-techniques based physical education occupied” (p. 21) after the English 1946 Education Act. This Act raised compulsory education to 15 years of age and boosted the development of the PE curriculum for “mass secondary education.” Before WW2 “women had dominated physical education teaching as a profession until the 1940s, but these post-war developments required the training of a large number of male physical educators” (p. 20): the extension of schooling included the puberty period, which is why “the dominant and deeply gendered form of physical education at this time, based on gymnastics and movement, made single-sex classes seem highly appropriate.” As Kirk accounts: “The men preferred a sports-based form of their field in contrast to the female-dominated gymnastics past, and a massive reconfiguration and reconstruction of school physical education was underway” (Kirk, 2017, p. 20).

To Ellen Singleton: “Games are such a large and integral part of the content of PE classes that any change in the pedagogical approach to games indicates changes to our shared educational philosophy about student’s needs – their methods of learning, their interests and attitudes, and their physical capacities” (Singleton, 2010, p. 27). This is the key question about any activity included in PE classes, and her narration of how games made their way into Canadian PE indicates that it was related to the Deweyan conception of play, “games, dance, and sports” in primary, while it was a chance for PE secondary teachers to “justify the introduction of more intense forms of competition, particularly into classes for males students” (p. 24). As a consequence: “Intense competitive team sports were, by the middle of the century, mainstay in American and Canadian males physical education programs […]. Over time, the emphasis on games in physical education has shifted from the question of whether games should be included in the curriculum, to questions about how games should be taught” (p. 25). Mauldon and Redfern’s experience seems mostly congruent: “The subject of Games is one which, as yet, has merited little genuinely serious attention. It is, of course, part of that plethora of multifarious activities which collectively are known as Physical Education, but whereas other major branches of this (notably dance, gymnastics, and swimming) have received certain amount of consideration in respect of education, the teaching of games continues in the main to be carried on along traditional and even stereotyped lines, with few questions asked as to the reasons for its inclusion in the curriculum” (Mauldon and Redfern, 1969, p. vi).

*Sport* is the product of the institutionalisation of games occurred in England in the nineteen century (During, 1984; Mangan, 1986), and it makes part of PE because they are motor activities whose practice can produce valuable effects on the multiple dimensions of personality. Even though, *ex post facto* argumentations that try to justify pedagogical decisions that never existed do not take into account that the same beneficial effects can be obtained with other kinds of activities. According to Parlebas (1986, p. 246), institutionalisation is mainly driven by the high economic value of sports competitions as spectacular sources of entertainment, but it is also linked to “foundational ethical imperatives” that seem today as unquestionable as ever: “Sport remains strangely associated with values which it does not respect as a mass phenomenon, but which help to maintain its positive image” (Parlebas, 1999, S:323) and stop many from making sticky questions: *Do we really need to play federation games most often? Do they have a higher educational value than traditional games?* (1978: Parlebas, 2017, p. 349–355).

The answer to this double question is a double no: “Federated games are not characterised by an uninterested educational richness, but by their value as spreaders of a certain power” (1978: Parlebas, 2017, p. 353), and sports prevail “because they are adult games that adjust to the powers with full command, and eventually to some of their counterpowers.” Even if, “analysing the sports phenomenon in terms of power does not necessarily imply condemning it: there is no society without an institution, nor an institution without power, and the gratuitous condemnation of sport is as ideological as its blind glorification.” The vindication of traditional games does not imply the vilification of sports, for it would be useless and unfair: today, as it was at the time, “it seems almost un-British to suggest that education could be complete without games!” (Mauldon and Redfern, 1969, p. 2), and no one with sound judgment should try to tackle a whole Empire: “This will be a perfect planet//only when the Game shall enter//every country, teaching millions//how to ask for Leg or Centre” (Gale, 1930, In Mangan, 2012). Should we?

### On the Consequences of Institutionalisation

The consequences of our including sports in PE curricula are the consequences of the institutionalisation of sports. In this sense, basketball, a game invented in 1891 by a PE teacher who got inspired by a traditional game, that was proposed in winter to a group of troublemakers as an alternative to the *boring* calisthenics, that in less than a decade spread all over America thanks to an educational institution, that got into the sports show-business by the turn of the century…, makes the perfect example (Naismith, 1941) to understand the intimate relationship between PE and sports. Basketball was such a successful *new game* because it abode perfectly by the sports model: a time-limited duel between two equal teams, quantitative and qualitatively speaking, that binds players together on a relational network based on constant loyalty and equal chances (Parlebas, 2002, p. 211). However, if human relationships in the real world can also be inconstant, unstable, and logically irrational, are sports the best way to learn about the others, about life in society, about the dialectical relationship between individual agency and collective systems? In other words: Can we really be convinced about the contribution of team sports to children’s socialisation (Parlebas, 1978a, 2017, p. 357)? Is sports socialisation really better than traditional socialisation (Etxebeste, 2012)?

Structurally speaking, sport is not perforce any better than traditional game. On the contrary, as far as socialisation is concerned, traditional games *put in play* human communication models far more diverse and enriching than sports:

All sporting games, whatever they may be, can be put at the service of authority, although traditional games, as a whole, are much less favourable than sport to the exercise of centralised and authoritarian power, for two main reasons. The first is that the rules of many traditional games propose uses of space, types of communication, criteria of success and possibilities of group decision that do not favour the establishment of an external, undisputed authority. The second is that the variety of situations generated by these games causes motor behaviours through extremely different exchange systems. A variety of this type, which responds to sometimes contradictory norms and attitudes, makes it difficult to unilaterally channel motor conducts towards a rigid system, towards a strict social shaping of the body (1978b: Parlebas, 2017, 354).

School sports practice can suppose the massive reproduction of just a few of the many relational configurations available, the “reproduction” (Bourdieu and Passeron, 1981) of exclusive-stable communication models that leave aside many other forms of antagonism and cooperation that offer interaction opportunities more suitable to dive into to the depths of human relationships.

**FIGURE 2:**
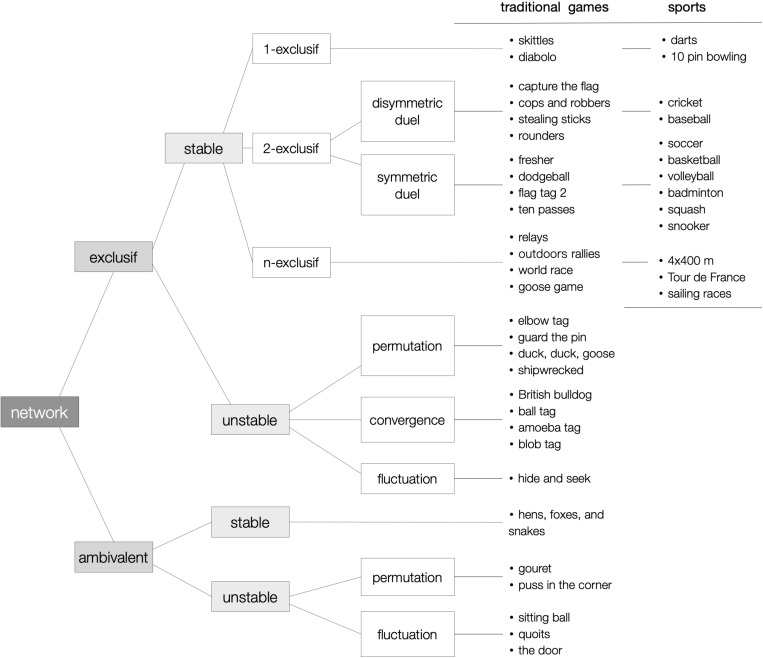
Networks of motor communications: comparison between sports and traditional games (modified from Parlebas, 1986, p. 238).

As shown above in Figure 2, according to their “motor communication networks” (Parlebas, 1999, R:26) and “network of changes of sociomotor roles” (R:50), sports are only present in four of the 10 categories of sporting games, and games in TGfU belong almost only to two of them: symmetric and dissymmetric duels. All sports’ communication networks are *n-exclusive* (any two participants cannot be at the same time partners and opponents), *stable* (the initial relationship between any two players is kept unchanged till the end of the competition), *complete* (in sociomotor networks there is always a positive or negative relationship between any two players, never neutral), and *balanced* (intra-team relationships are always positive and inter-team relationships always negative) (Parlebas, 1986, p. 235). Conversely, traditional games accept *instability* (alliances, friendship, and antagonism that change along the time) and *ambivalence* (uncertainty about the others’ real intentions to help or harm one’s interests), making possible for the players to enjoy the relational possibilities offered by playing situations not fully predetermined by outer institutions.

Many tagging games possess exclusive-unstable networks with three role-changing possibilities: *permutation* of roles between the two players involved in a catch; *convergence* of all tagged players into the chasing role; and *fluctuation* of roles due to role changing without a strict order. Many other games, those which Parlebas (2010b; 2011a) likes to call *paradoxical*, give room for even more liberty to choose: all the fun in a game as simple as “puss in the corner” comes from the choice that any player on a corner can make to be loyal to or betray the *comrades* on the other corners. Can anybody imagine a better way to express oneself than a situation in which any decision is a declaration of hate or love? In this sense, can there be any better way to learn *simple* moral principles than putting them at play? There is no doubt that sports can offer remarkable educational experiences, but from a pedagogical point of view their practice comes along with a double jeopardy we must be aware of from the pedagogical angle: that of reducing action to movement, and that of reducing interaction to obedience. What a challenge!

## On Traditional Games, Understanding, and Teaching as Communication

Traditional sporting games have helped us make our point about two issues on which later TGfU seems less interested than the *founding fathers*: the nature and classification of motor games, and the educational consequences of disregarding sporting games other than sports. The inquiry on efficiency in games teaching received much attention in the mid twentieth century, not only in France and Britain: for instance, Mahlo (1969), from the German Democratic Republic, developed a remarkable work on the “tactical act” for 6- to 10-year-old socialist students greatly based on the psychologist Sergei Rubinstein’s ideas. Thorpe et al. (1986) realised that technical conception of games and teaching is detrimental to the legitimate aspirations of physical educationists and the best interest of pupils, that a call should be made “away from a skills-based lesson and towards a more cognitively based approach” (5). Almost two decades earlier, Parlebas defended the same in his first paper: “Philosophical training has familiarised physical education teachers with the universe of meanings, symbols and values, empowering them to not just be technicians or lessons providers; it has taught them to be independent of stereotyped structures by showing the dangers of surrendering to banal techniques, however magnificent they might be” (1959: Parlebas, 2017).

In 1967, Allen Wade, Football Association’s Director of Coaching, published a book that, as we know, was revolutionary and inspiring: *The F.A. guide to training and coaching* (Wade, 1979). Written for teachers and coaches alike, it was promoted as follows “The theme of the book takes full account of modern tactical development and emphasises the need for the modern footballer to ‘read the game’ and understand the systems and tactics that are outlined.” There are radical differences between our conceptual model of game-playing and Wade’s analysis based on the dyad technique/skill, but his references to *common factors, unpredictability, interference of intentions, cooperation and opposition, and decision making* (1967: Wade, 1979, p. 180) are an outstanding prove of wisdom and strength for what TGfU would become. In actual fact, the interest gained by the “sports-coaching turn” (Kirk, 2017, p. 19) is far from being inadequate nor unexpected: it is the cornerstone of our double story, because any general proposal for school PE ignoring the internal logic of teaching situations is doomed to grow on wasted lands.

### On Understanding as Semiosis

As Wade (1979) pointed out, unpredictability, decision-making, collaboration, opposition, and interference of intentions are the key elements of football-playing, because football and all team duels belong to the “motor action domain” (Parlebas, 1999, D:74) in which *collaboration and opposition* occur at the same time. It is the presence of opponents what willy-nilly generates on the players decisional uncertainty no matter their competence. From the learning side, we conceive understanding as the competence to put in play the principles of game-playing associated with the “internal logic” (Parlebas, 1999, L:4) that emerges from the interpretation of the game’s rules; from the teaching side, we conceive understanding as the competence to assess and increase the level of clarity that the players’ conducts show in relation to the internal logic of the situation. From both sides, “uncertainty: Property of unpredictability attached to certain elements of a situation” (Parlebas, 1999, I:1), is the most imperative praxic consequence of the game system, for it determines how games must be solved and how players must be trained in terms of *decision-making*. Generally speaking, the two sources of uncertainty in sporting games, namely, “social” and “spatial,” can be operationalised with three binary variables that inform about the presence or absence of uncertainty due to the relationships with “partners,” “opponents,” or “space.” The combination of these tree dichotomic traits results in a classification with eight motor action domains that, individually, propose equivalent experiences, and collectively allow to understand and manage the total of ludomotor activities (Parlebas, 1999, C:1).

TGfU is mostly about *teaching to understand sports opposition* in the form 2-exclusive-stable communication networks networks, that is, duels of teams or individuals: only one of Elli’s eight categories (Ellis, 1983), concretely “unopposed target games” like bowling and golf, lacks opposition, and Almond (1986) does not even make such a distinction in his “target games” category. We have seen above (Figure 2) that sporting duels are only two of the many cases in sporting games, but it is also true they constitute a subset of motor tasks in which *instrumental praxic communication* reigns: in sporting duels motor interaction is purely strategical, driven by the scoring system, and built semiotically upon the bodily procedures permitted by the rules. Goffman’s description of “strategic interaction” is a remarkable account of how antagonising, deceptive interaction works: “In every social situation we can find a sense in which one participant will be an observer with something to gain from assessing expressions, and another will be a subject with something to gain from manipulating this process. A single structure of contingencies can be found in this regard which renders agents a little us all and all of us a little like agents” (Goffman, 1969, p. 81). Although he did not look into their semiotic grounds, his analysis of the so-called “expression games” is as inspiring as accurate his distinction of *unwitting, naïve, covering, uncovering and counter-uncovering* moves.

Peirce defined “semiosis” as *the action of signs*, and Parlebas chose “semiotics” (Saussurean semiology actually) as the cornerstone of his epistemology: “Sport is a world of signs: of signs, not of stimuli. Is it possible to carry on treating players in action as they were stimulus-response mechanisms? Is it possible to be content with analysing their acts from the Pavlovian model of conditioning? Sporting game is a place riddled with immediate, literally *embodied* meanings: each motor behaviour carries a meaning that the other participants must interpret to act appropriately. Sitting-ball players, like basketball players, try to extract tactical meanings from the acts that interweave before them” (Parlebas, 2017, p. 277). “Semiotricity: field and nature of motor situations considered from the angle of the use of sign systems directly related to the participants’ motor conducts” (Parlebas, 1999, S:43), really puts “meaning” in the centre of teaching, learning, and research, allowing to outpace mechanistic, dualistic conceptions of PE, in the first place, and dualistic, technique-based sports coaching at any level of analysis, as a consequence. This semiotic nature of motor conducts makes them the cornerstone of PE, therefore understood as the *pedagogy of motor conducts* (E:11), and casts off any reductionist, dualist conceptions of human beings: “The term ‘movement,’ so often invoked in physical education still today, is notoriously inadequate and the prove of that old conception, which takes into account the product and not the producing agent. The notion of movement refers to the idea of a bio-mechanical body defined by displacements observed from the outside; it is somewhat concerned with describing gestural ‘utterances’ from which the subject is excluded as such and whose culmination is ‘the’ technique, the abstract and depersonalised gestural model” (C:108).

From this angle, any player’s “motor conduct: meaningful organisation of motor behaviours” (Parlebas, 1999, C:105) can be analysed as a sign whose *signifier* is the observed motor behaviour, and whose *signified* is the tactical, relational, or referential sense. The semiotor logic of team sporting games is essentially different from the “natural semiotricity” of outdoor pursuits, the “referential semiotricity” of body expression and mime, and the “socio-affective semiotricity” of paradoxical games (S:43), but they all share the same semiotic, cognitive structure. Motor conducts are bodily expressions of a personality in endless interaction with their vital circumstances, signs that can also be interpreted by competent teachers as *a level of competence, a learning outcome, a trait of character, emotional state, relational status, tactical decision, etc*. Nonetheless, the competitive logic of sports *forces* to take them as “motor decisions” (D:5) to be assessed in terms of strategical and tactical efficacies. Semiotor angle is the only valid perspective to address the metacommunicative nature of sports interaction: a game establishes a normative layer of direct motor communication that regulates the material limits of interaction (i.e., tackling, charging, passing, hitting, shooting, etc.), on top of which the players’ intentions are built and evolve. This “indirect praxic communication” (Parlebas, 1999, C:65) is a battle of signs called “praxems” (P:26) through which individuals and teams try to outwit their adversaries in a constant game of guesses, deceptions, and make-beliefs. This process, far from being magic, can be traced and trained from very young ages, like in football (Oboeuf et al., 2019).

*Understanding* is best, if not only, conceived as the semiotic performance that allows players to infer and interfere with other players’ intentions on a bodily basis, and *teaching* is best conceived as a thoughtful process of building up semiotor habits that provides the players with the competence to anticipate and pre-act efficiently on pitches and courts where everybody can be fooled. Understanding is interpreting, and interpretation is the outcome that results in “motor conducts” as far as an individual agent is concerned, and in “motor action” as far as the whole situation is concerned. Any motor behaviour, or articulation of motor behaviours, is a Peircean “motor interpretant” (Martínez-Santos, 2007) that participates in a triadic, indexical relationship with the rest of the motor behaviours of the situation. Furthermore, *social uncertainty* and *sociomotor intelligence* are bound together by the essential randomness attached to subjects; the only certainty attached to opposition games that Koppet (1973) so wisely identified in basketball is that “the essence of the game is deception”: the only way to learn to play these games is by understanding that signs never lie, but opponents always try to.

### On Teaching as Communication

It is also a tradition to propose traditional games to teach sports. So did Sleap (1984) for mini-sports, and Mahlo (1969) for the tactical act, who surprisingly made compatible disdain and appreciation for them when saying that the relatively simple “little games” prepare for team sports: “A qualitatively superior mean of development and physical education, and an important form of cultural activity of human society” (p. 149). However, team sports, such as football, basketball, and handball, make a perfect *intervention strategy* for a teaching traditional sporting games, and teachers can increase its beneficial effect by using an adequate *teaching style for understanding*. This is what Parlebas and Dugas (1998) were able to prove through a fully controlled piece of experimental research. “Learning transfer: the effect that can be observed when the execution of an activity modifies, positively or negatively, the accomplishment of a new activity or the reproduction of an old one” (Parlebas, 1999, T:90) is the cornerstone of teaching and PE, understood as an after-effects searching, deliberate motor intervention practice.

Teaching can be conceived as a communicative process between teacher and learner through practice tasks (Martínez-Santos, 2018): teacher and learner are indirectly connected through the internal logic of the exercises selected by the former, and directly connected by the logic of face-to-face communication between them, as explained by Poyatos (1994). Taken two by two, these three elements (namely, “teacher,” “learner,” and practice “task”) generate the three basic dimensions of any intervention process: the design and selection of tasks, that is, the *strategic dimension*, corresponds to the axis “teacher-task”; the *praxic dimension* corresponds to the motor action the emerges from the axis “task-learner”; the axis “learner–teacher” corresponds to the *stylistic dimension*, the many ways in which educational instruction can be managed. In this vein, TGfU may seem more a style-based proposal, whereas praxeological proposal primarily strategic, but this could be an illusion due to its presentation as a six-ordered-phase model of teaching (Bunker and Thorpe, 1982) and its illustration with teacher–student dialogues (Almond, 1986). Even so, this would not be as bad as the “illusion [that] haunts stadiums and gyms: educators tend to think that the nature of the activities matters little, that it is just a simple means at the service of the chosen educational purposes, that teachers have full powers over them. In fact, the master of the game is not the teacher, but the game: a system of rules that imposes its dictates; a system that has its own logic and defines the universe of actions and permitted conducts” (1979: Parlebas, 2017, p. 383).

Far from any *nature* vs. *nurture* kind of battle, but as close to didactical *falsifiability* as possible, we can be certain that the absence in practice of key aspects of the games makes developing the targeted competences most unlikely. Twenty years ago McMorris concluded: “With regard to which method [TGfU or technical] is the better for teaching games, the evidence is inconclusive and much more research is necessary. The research into net games suggests that neither method is more successful than the other for those activities. For team games the lack of research makes it impossible to make a definitive statement” (McMorris, 1998, p. 70). We feel concerned too by his conclusions: “Overall it could be argued that TGfU has directly provided little or nothing new to the motor learning literature. […] However, specificity of practice should not be ignored and modifying in TGfU style, e.g., playing ‘hockey’ without sticks, violates specificity and could lead to negative transfer of training when the real game is introduced […] The decision as to which method the teacher adopts is more a philosophical one than one based on empirical evidence” (p. 71).

**FIGURE 3:**
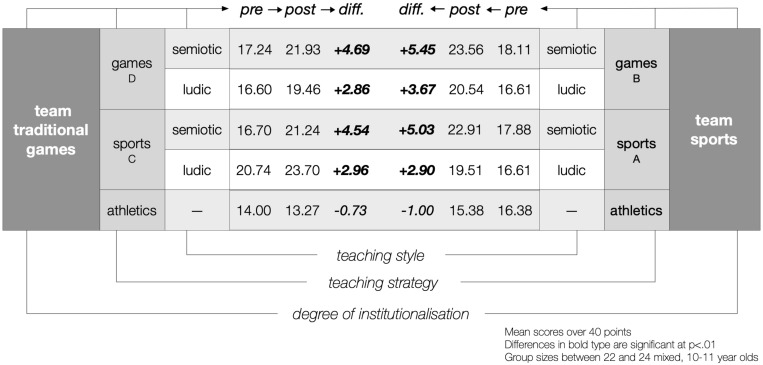
Learning transfer, traditional games, and sports (Dugas, 2001).

Peirce could hardly agree with McMorris’ last statement. According to his pragmatist maxim, the highest level of clarity can only be attained by putting concepts like teaching and transfer to the test of learning outcomes’ reality. On our part, we must disagree with McMorris because Parlebas and Dugas have provided us of empirical evidence that the content of practice matters (Figure 3): athletics is not the basis of all sports, and internal and external logics of games are independent in terms of learning transfer (Parlebas and Dugas, 1998; Dugas, 2001, 2006, 2010). A classic experimental design, with full control over the grouping (i.e., age, gender, sports experience, academic level) and didactical (teaching strategy and style) variables, allowed them to pre-test and post-test the sociomotor competence in traditional and institutionalised team games after eight sessions practising traditional games, sports, or athletics (control groups). The results are crystal clear: firstly, structural similarity between team duels produced positive learning outcome in all groups (A–D), regardless of whether they were tested in sports (A and B) or traditional games (C and D), regardless of whether practice consisted of sports (A and C) or traditional games (B and D); secondly, the practice of psychomotor activities (athletics) produced no change on the sociomotor competence associated with playing team games; and, finally, the *teaching for understanding*, semiotor style produced higher learning levels than a ludic one in all experimental groups (A_semiotic_ > A_ludic_; B_semiotic_ > B_ludic_; C_semiotic_ > C_ludic_; and D_semiotic_ > D_ludic_). Even more, the structure of praxemic interaction, which is the core of the motor competence of teams sports, is the best scaffolding for teachers and coaches to teach players: to be aware of their own intentions, to recognise the playing circumstances, and to articulate their conducts with the other players’ intentions (Martínez-Santos, 2007).

In brief, these results are most valuable for three reasons: firstly, they prove that the internal logic of educational activities overcomes the external features of their practice, for example social recognition or economic value; secondly, they show that the internal features of the tasks created by the rules determine the limits of sociomotor learning transfer; and finally, they reinforce the belief that there exists such a thing as *teaching for understanding*. As we said, TGfU seems to lean more on teaching-style than on teaching-strategy, although Almond took great advantage of the distinction that Suits made between games’ primary rules (constitutive) and secondary rules (operational) to help his students in games making (Harvey et al., 2018, p. 170). Besides, their remarks on the *praxic consequences* of rules modifications (Thorpe et al., 1986) are the very essence of the strategic dimension of teaching based on the praxeological modification of games. We already said that this is a tale about two perspectives *driven on parallel tracks for too long* despite the overwhelming quantity of coincidences.

## One Tale, Two Stories, and Three Conclusion

Traditional games can produce the same kind of learning outcomes than sports, and there is no difference between their practice regarding the benefits of a semiotic, understanding style of teaching. The development of *thinking players* cannot be an educational objective, but the development of *thinking citizens* can be better attained through the development of the intelligence associated with sociomotor action, a venture in which traditional games have a world to offer. Evidently, we have no answer to the question we asked ourselves in the first place: *Why are traditional games absent from the TGfU rationale?* but we truly believe that TGfU advocates and practitioners can agree with us that traditional games have an immense educational value that should not be taken for granted. The *what*, the *how*, and the *why* of games in PE are better understood if we take traditional games as a contrast, as an option, and as a choice: as a contrast, to notice that sports, that is, institutionalised games, are nothing but a subset of physical games and activities; as an option when it comes to teaching sports, a venture in which traditional games offer outstanding, most transferable learning opportunities; and as a choice, because the structural variability that traditional games offer is remarkable from an educational, cultural point of view.

Teaching-games-for-understanding and motor praxeology represent two different solutions to the scientific-foundations-of-PE problem, although we feel enforced to contest Peter Watson, who asserts in his superb intellectual history of the twentieth century: “Many continental thinkers, especially French and from the German-speaking lands, were devoted to the marriage of Freud and Marx, one of the main intellectual preoccupations of the century, and maybe the biggest dead end, or folly, which affected, in France most of all, of blinding thinkers to the advances in the *harder* sciences. This has created a cultural divide in intellectual terms between francophone and anglophone thought” (Watson, 2001, p. 753). True as it may be, for one of the most beautiful texts by Parlebas – *Sporting game, dream and fantasy* (Parlebas, 1975) – is built upon the Freudian triad *Id*, *ego*, *and super-ego*, it is also true that he very much looks like a *classic scientist* in the Foucauldian sense (Foucault, 1991, p. 7), for his relentless search for mathematical formalisation and conceptual clarification of sporting games and motor action (Martínez-Santos, 2017).

McMorris eventually accepts that TGfU raises “a number of very important issues that motor learning discipline needs to address” (McMorris, 1998, p. 71), like ecological validity, task complexity and transfer, implicit learning, and transfer of games’ principles. However, in doing so, maybe unintentionally, McMorris also reinforces the serfdom so many in the Academy expect from physical educationists. The academic world is made of *fundamental sciences* and self-proclaimed fundamental scientists like anatomists, physiologists, phycologists, sociologists, philosophers, historians, etc., from whom teachers, trainers, coaches, sports monitors, etc., all of them devoted practitioners, must learn and apply concepts, theories, and evidence. TGfU has received critics for its alleged lack of theoretical soundness (Harvey et al., 2018), but it could be argued that none of its *relatives* (Butler et al., 2008) or *contenders* (Renshaw et al., 2015; Jarrett and Harvey, 2016) has shown either much interest in developing an autonomous, specific understanding of what sporting games playing involves from the agent’s perspective. We believe that *understanding* in TGfU and game-based approaches still remains unexplored while admitting, at the same time, that TGfU can perfectly hold out against critics and gain fundamental conceptual insight if opened up to semiotor perspective and traditional games.

In this sense, our last conclusion is that TGfU and game-based approaches can take great advantage of the motor-praxeological rationale for three key reasons: firstly, because concepts like understanding, game sense, and action principles are operatively conceived, that is, semiotically linked to the reality of the playing process; secondly, because the inner structures of the games that constrain players and guide their motor conducts permit to integrate team sports in the general system of sporting games, no matter their level of institutionalisation; and, finally, because teaching practice is better thought of and more systematically developed upon the operational concepts of internal logic, expected outcomes of game playing, and teaching style. This time, unlike in Dickens’ novel, Paris could be the place to go to in search of solutions, not the city to run away from in hope of consolation.

## Author Contributions

RM-S wrote and edited the text and produced the figures. All authors have equally taken part in the design, literature review, and discussion of the drafts, making valuable contributions along the whole process.

## Conflict of Interest

The authors declare that the research was conducted in the absence of any commercial or financial relationships that could be construed as a potential conflict of interest.

## Footnotes

1 This book is online at https://books.openedition.org/insep/1067; the reason why all references will be related to this online edition, and any citation, will be identified by the initial letter of the term in French, the chapter it is included in, and the ordinal number of the paragraph: L:56 is the 56th paragraph in chapter L.
